# Prospective Evaluation of Ultrasonic Surgical Dissectors in Hepatic Resection: A Cooperative Multicenter Study

**DOI:** 10.1155/1992/59508

**Published:** 1992

**Authors:** Bertrand Millat, Jean-Marie Hay, Bernard Descottes, Abe Fingerhut, Pierre-Louis Fagniez

**Affiliations:** 1 Hôpital Antoine Béclère Clamart France; 2 Hôpital Louis Mourier Colombes France; 3 Hôpiml Dupuytren Limoges France; 4 Centre Hospitalier Intercommunal 10 rue de Champ Gaillard Poissy Cedex 78303 France; 5 Hôpital Henry Mondor Creteil France

## Abstract

Blood loss is the major cause of postoperative mortality and morbidity associated with hepatic resection. A prospective multicenter study was conducted to determine if ultrasonic dissectors (USD) were useful in hepatic resection and could reduce this hemorrhagic risk. Forty-seven hepatic resections were performed in 42 consecutive patients during a two month period in 11 public, surgical centers. Twenty-one patients had primary or secondary malignancies, six had benign tumors, two had biliary cysts, one had cholangiocarcinoma, one had Caroli’s disease, and 11 had hydatid cysts of the liver. Two different USD devices were evaluated (CUSA System-Lasersonics and NIIC-DX 101 T). The hepatic resections tested included a wide range of procedures. Each surgeon had the possibility of choosing between the USD and his own usual technique for each operative step and according to local conditions. The average volume of blood infused, irrespective of the underlying pathology or the procedure performed, was 1.0 L (range 0-4.8 L). Fourteen patients required no transfusions. No operative or immediate postoperative deaths were recorded. Five major complications, all unrelated to the use of the USD, developed in three patients. Access to intra and extraparenchymal arterial and venous tributaries and particularly the control of the hepatic veins were facilitated by USD. While transection of hepatic parenchyma was neither easier nor faster than with conventional techniques, it was found to be less hemorrhagic. Overall appraisal was expressed on an analog scale; the USD was found to be helpful or very helpful in 75 percent of all resections. With regard to the pathology being treated, total or partial excision of hydatid cysts was greatly enhanced by the use of the USD while this benefit was not found for wedge resections of other hepatic lesions. With regard to user friendliness and maintenance, the NIIC-DX 101 T device was preferred. We conclude that the USD facilitates formal hepatic resections. Converging opinions emerging from various surgical centers reinforce this conclusion.


*HPB Surgery*, 1992, Vol. 5, pp. 135–145
Reprints available directly from the publisher
Photocopying permitted by license only

© Harwood Academic Publishers GmbH
Printed in the United Kingdom

### PROSPECTIVE EVALUATION OF ULTRASONIC SURGICAL DISSECTORS IN HEPATIC RESECTION : A COOPERATIVE MULTICENTER STUDY

BERTRAND MILLAT

*Hôpital Antoine Béclère, Clamart, France*

JEAN-MARIE HAY
*Hôpital Louis Mourier, Colombes, France*

BERNARD DESCOTTES
*Hôpital Dupuytren, Limoges, France*

ABE FINGERHUT
*Centre Hospitalier Intercommunal, Poissy, France*

PIERRE-LOUIS FAGNIEZ
*Hôpital Henry Mondor, Creteil, France*

*(Received 8 August 1991)*

Blood loss is the major cause of postoperative mortality and morbidity associated with hepatic resection. A prospective multicenter study was conducted to determine if ultrasonic dissectors (USD) were useful in hepatic resection and could reduce this hemorrhagic risk. Forty-seven hepatic resections were performed in 42 consecutive patients during a two month period in 11 public, surgical centers. Twenty-one patients had primary or secondary malignancies, six had benign tumors, two had biliary cysts, one had cholangiocarcinoma, one had Caroli's disease, and 11 had hydatid cysts of the liver. Two different USD devices were evaluated (CUSA System-Lasersonics and NIIC-DX 101 T). The hepatic resections tested included a wide range of procedures. Each surgeon had the possibility of choosing between the USD and his own usual technique for each operative step and according to local conditions. The average volume of blood infused, irrespective of the underlying pathology or the procedure performed, was 1.0 L (range 0-4.8 L). Fourteen patients required no transfusions. No operative or immediate postoperative deaths were recorded. Five major complications, all unrelated to the use of the USD, developed in three patients. Access to intra and extraparenchymal arterial and venous tributaries and particularly the control of the hepatic veins were facilitated by USD. While transection of hepatic parenchyma was neither easier nor faster than with conventional techniques, it was found to be less hemorrhagic. Overall appraisal was expressed on an analog scale; the USD was found to be helpful or very helpful in 75 percent of all resections. With regard to the pathology being treated, total or partial excision of hydatid cysts was greatly enhanced by the use of the USD while this benefit was not found for wedge resections of other hepatic lesions. With regard to user friendliness and maintenance, the NIIC-DX 101 T device was preferred. We conclude that the USD facilitates formal hepatic resections. Converging opinions emerging from various surgical centers reinforce this conclusion.

KEY WORDS: Hepatic resection, ultrasonic dissectors

---

Address correspondence to: Abe Fingerhut, Centre Hospitalier Intercommunal, 10 rue de Champ Gaillard, 78303-Poissy Cedex, France

### INTRODUCTION

Blood loss is the major cause of postoperative mortality and morbidity associated with hepatic resection[1–4]. With increasing interest in liver resection for the removal of tumors[4–6], new technologies have been proposed as aids, such as the microwave tissue coagulator[7], ND:YAG laser[8], water jet[9], and ultrasonic dissectors (USD)[10, 11]. Experimental and clinical results showing that USD can reduce the hemorrhagic risk have already been published[10–14]. Because of relatively high costs, however, we thought that a large scale trial should be undertaken before advocating widespread use of the USD. A prospective multicenter study was therefore conducted to determine whether and under what circumstances the USD would be useful. In addition, the operative performances and problems of maintenance of the two dissectors available at the time of the study were compared.

### METHODS

### *Patients*

Forty-two consecutive patients with indications for elective hepatic resection were operated on during the eight week period of the study in 11 public surgical centers of three French Associations for Surgical Research (Association for Surgical Research, University Association for Surgical Research and Assistance Publique Surgical Association for Medical Evaluation). There were 23 male and 19 female patients, whose mean age was 50 years (range 22–79 years). All patients with hepatic lesions above 18 years of age were eligible for the study irrespective of the underlying pathology or the type of lesion and resection performed. None of the patients seen within the time interval were withheld. At least two patients were operated on in each center, and all operations were performed by senior surgeons familiar with hepatic surgery. Hepatic tumors, more often malignant ($n=21$) than benign ($n=6$), were the most common indications. Malignant tumors were more often secondary ($n=16$) than primary ($n=5$). Hydatid cysts were the second most common indication ($n=10$). Two patients were operated on for complicated biliary cysts, one for cholangiocarcinoma of the porta hepatis with spread to the adjacent hepatic parenchyma, and one patient for Caroli's disease.

### *Assessment criteria*

Questionnaires were specifically drawn up for the study and were filled out by the participating surgeons in a prospective manner. Although the USD was used in all operations, the surgeon was free to choose between the USD and his own usual technique for each individual step of dissection according to operative circumstances. For a given operation, only one of the two apparatus was available. The main end point of the study was the quantity of blood and total fluid volumes infused during the operation. Subsidiary criteria were: (1) operative mortality (deaths occurring during the first postoperative month), and (2) postoperative morbidity (hemorrhage, biliary leaks, intra-abdominal sepsis).

Specific criteria were used to evaluate the USD: (1) capability of the USD to enhance the dissection of the main extra or intraparenchymal vascular pedicles

(hepatic artery, portal and hepatic veins); (2) evaluation of the ease, rapidity, and degree of bleeding of parenchymal section as compared with usual techniques (cautery, finger or clamp fracture); (3) operative incidents related to the use of the USD; (4) evaluation at the end of the operation of the usefulness of the device on an analog scale graded from zero (the USD is useless) to five (the operation could not have been performed without it); (5) usefulness of the USD according to the type of hepatic resection performed.

The duration of operation was measured from the time of incision until skin closure. The duration of resection was measured from the start of dissection until complete achievement of parenchymal hemostasis.

In addition, each surgeon was asked to express his opinion about the ease of utilization, the capability, and the handiness of the hand-piece. The operating room staff was asked to evaluate the general design, ease of set-up, training for utilization, transportation, upkeep, and maintenance of the device.

### *Surgical Operations*

The abdomen was entered through a right ($n=13$) or bilateral ($n=29$) subcostal incision. Thirty-seven patients had a single hepatic resection whereas five had multiple resections. The techniques of major or minor hepatic resection performed with the USD have been reported elsewhere[11]. The type of operation performed with regard to underlying disease is reported in Table 1. Pringle's maneuvre was performed in four cases; total vascular exclusion of the liver was never used. The hepatic parenchyma surrounding the tumor was usually normal; in five cases it was altered by infection, fatty infiltration or hyperplastic nodules and in three cases, by cirrhosis. The raw hepatic parenchymal surface exposed after resection was estimated at a mean of 95 $cm^2$ (range 12–375 $cm^2$). In ten patients the postresection surface was covered by omentum and in 29 cases, by various local hemostatic substances. No additional procedure was used to cover the raw surface of the liver in 14 cases.

### *Devices*

Two different USD were evaluated: the Cavitron Ultrasonic Surgical Aspirator

**Table 1** Type of resection performed according to the initial disease

| *Method* | *Malignant Primary Tumors* | *Secondary Tumors* | *Bening Tumors* | *Hydatid Cysts* | *Biliary Cysts* | *Others** |
|---|---|---|---|---|---|---|
| Lobectomy | 1 | 6 | 1 | 1 | 0 | 1 |
| Left lateral segmentectomy | 1 | 2 | 1 | 3 | 0 | 1 |
| Other segmentectomies | 2 | 3 | 2 | 0 | 0 | 0 |
| Wedge resection | 1 | 7 | 2 | 0 | 0 | 0 |
| Cyst excision | — | — | — | 10 | 2 | — |
| Total resections | 5 | 18 | 6 | 14 | 2 | 2 |
| Total patients | 5 | 16 | 6 | 11 | 2 | 2 |

* One case of cholangiocarcinoma, one of Caroli's disease

(CUSA system; Lasersonics) and the Ultrasonic Surgical System DX-101 T (NIIC; Baxter). The USD consists of a gas sterilizable lightweight hand-piece connected to a portable control and power console by a 450 cm sterile cable. The pencil-grip surgical hand-piece is separable from the rest of the apparatus and contains a magnetostrictive (CUSA System) or electrostrictive (NIIC-DX 101 T) transducer that converts electrical energy into mechanical motion. The transducer oscillates longitudinally at 23 kHz (CUSA System) or 28 kHz (NIIC-DX 101 T) and is connected to a hollow conical interchangeable titanium tip. This results in a longitudinal tip excursion of 300 $\mu$m (CUSA System) or 250 $\mu$m (NIIC-DX 101 T). When the tip comes into contact with the target parenchyma, these vibrations create cellular fragmentation and disruption which are proportional to the water content of the tissues. The greater the water content, the more easily the tissue is fragmented. At lower settings, collagen and elastin fibers are spared so that blood vessels and bile ducts can be identified, isolated, and subsequently controlled by cautery, hemostatic clips, ligation, or suture. Irrigation over the tip prevents excessive heating and facilitates the aspiration of fragmented tissue. An operating room technician sets the instrument up and controls the ultrasonic power available in the hand-piece and the irrigation-aspiration rates from a remote console.

### RESULTS

Overall, irrespective of the pathology or procedure performed, the average volume of blood and other fluids infused during the operation was 5.4 ± 3.8 L. The volumes of blood, plasma, and other fluids infused during the operation according to the type of hepatic resection performed are listed in Table 2. The volumes infused for malignant and benign tumors were 6.8 ± 4.5 L and 4.1 ± 2.2 L, respectively; volumes were 5.5 ± 3.3 L for resections of secondary colorectal metastases and 3.3 ± 1.5 L for total or partial excisions of hydatid cysts. The average volume of blood infused during the twelve cyst excisions was 0.4 (0-1.0) L; two of the twelve patients had multiple resections; eight of the ten patients who had cyst excisions only required no blood infusion at all during the operation.

**Table 2** Volumes (L) of blood, plasma, and other fluids *infused during the operation according to the type of hepatectomy performed

| *Types of resection* | *No* | *Blood* | *Plasma* | *Other fluids* | *Total* |
|---|---|---|---|---|---|
| Lobectomy | 10 | 1.9<br>(0.5–4.8) | 3.0<br>(0.6–7.2) | 3.8<br>(1.5–6.5) | 8.7<br>(3.8–15.5) |
| Left lateral segmentectomy | 8 | 1.1<br>(0.0–2.7) | 1.4<br>(0.0–2.4) | 3.0<br>(1.5–5.0) | 5.5<br>(1.8–7.3) |
| Other segmentectomies | 7 | 0.8<br>(0.0–2.4) | 0.9<br>(0.0–2.1) | 2.5<br>(2.0–4.2) | 4.2<br>(2.0–7.5) |
| Wedge resection | 10 | 0.9<br>(0.0–2.7) | 0.8<br>(0.0–2.4) | 3.0<br>(1.0–11.0) | 4.7<br>(1.0–14.3) |
| Cyst excision | 12 | 0.4<br>(0.0–1) | 0.5<br>(0.0–1.6) | 2.6·<br>)1.5–3.5) | 3.5<br>(1.5–4.3) |
| All resections | 47 | 1.0 | 1.2 | 3.0 | 5.4 |

* mean (range)

None of the 42 patients died intraoperatively. Two deaths (4.7%) were recorded during the same hospitalization, both due to liver failure with ascites. One patient died ten days following a wedge resection for a hepatocellular carcinoma whereas the second patient died two months after an extended right lobectomy for multiple metastases secondary to colonic carcinoma. Five patients (12%) had major complications. One patient had an intra-abdominal abscess requiring surgical drainage. One patient sustained postoperative hemorrhage originating from the abdominal wound. Three patients had biliary tract leaks. One healed spontaneously. The second patient had a biliary leak related to intraoperative left duct injury. The third patient, who presented with cholangiocarcinoma of the porta hepatis, had undergone preoperative percutaneous transhepatic biliary drainage; he developed an intra-abdominal abscess requiring surgical drainage as a result of bile leakage along the catheter track after the catheter was removed. There were no local complications in the remaining 35 patients (83%).

With respect to other possible techniques, the USD was elected by the surgeon for intra or extraparenchymal dissection of the biliary ducts in 47% of resections, in 28% of cases for dissection the hepatic artery, in 38% for the portal vein, in 64% for the hepatic veins, and in 11% for the inferior vena cava. When the dissector was used, the surgeons considered it advantageous in 92%, 100%, and 73% of cases for arterial, portal, and biliary duct dissection, respectively. One left hepatic duct injury related to the USD was recorded: this was repaired immediately without subsequent problems. Dissection of the hepatic veins and of the inferior vena cava was thought to be easier with the USD in 95% and 100% of cases, respectively. One case of accessory hepatic vein injury, unrelated to the use of USD, was mentioned.

Ultrasonic dissectors were used for parenchymal section in all cases but one. The comparison between USD and conventional methods (fracture techniques or electric cautery) for parenchymal section is recorded in Table 3. Ultrasonic dissectors were thought to reduce blood loss but made the hepatic resection neither easier nor more expedient. At the end of the operation, the surgeon made an overall subjective evaluation of the usefulness of USD taking into account both the dissection of the main pedicles and the transection of hepatic parenchyma. Even though no one operator thought that the USD was indispensable (grade 5), 73% believed it to be helpful (grade 3) or very helpful (grade 4) (Figure 1). The benefits derived from the use of the USD varied according to the type of procedure performed (Table 4). Major hepatectomies and segmental resections were greatly enhanced by the use of USD as compared with the other types of resection. The overall assessment showed no difference between left lateral segmentectomies, atypical resections performed for removal of secondary metastatic deposits, and excisions for hydatid or biliary cysts. Nevertheless, the benefits derived from the use of USD in parenchymal section were not similar for the two last procedures. With respect to fracture techniques or electrocautery, 70% of the surgeons favored the USD for excisions of cysts and only 30% for the wedge hepatic resection of metastatic tumors.

The time required for operation and hepatic resection is found in Table 5. The performances were similar with the two devices. On the other hand, the ease of utilization, the handiness, and maintenance of the two devices were not identical. Differences were essentially related to the characteristics of the handpiece. Greater weight and the presence of suction tubes made the CUSA handpiece System less

**Table 3** Comparison between ultrasonic dissectors (USD) and conventional methods for parenchymal section

| | *Opinions (%) on USD as compared to fracture technique* | | | *Opinions (%) on USD as compared to electrocautery* | | |
|---|---|---|---|---|---|---|
| | *More* | *Equal* | *Less* | *More* | *Equal* | *Less* |
| Easiness | 32% | 54% | 14% | 29% | 61% | 10% |
| Rapidity | 10% | 12% | 78% | 5% | 27% | 68% |
| Bleeding control | 10% | 20% | 70% | 5% | 29% | 66% |

**Table 4** Surgeons' evaluation at the end of the operation on an analog scale grading from 0 to 5 of overall ultrasonic dissector usefulness. The results are expressed according to the type of procedure performed*

| *Types of resection* | *No of cases* | *Analog Scale* |
|---|---|---|
| Lobectomy | 10 | 3.6±0.9 |
| Left lateral segmentectomy | 8 | 2.7±0.8 |
| Other segmentectomies | 7 | 3.3±1.6 |
| Wedge resection | 10 | 2.7±1.1 |
| Cyst excision | 12 | 2.8±1.3 |

* mean ± SD.

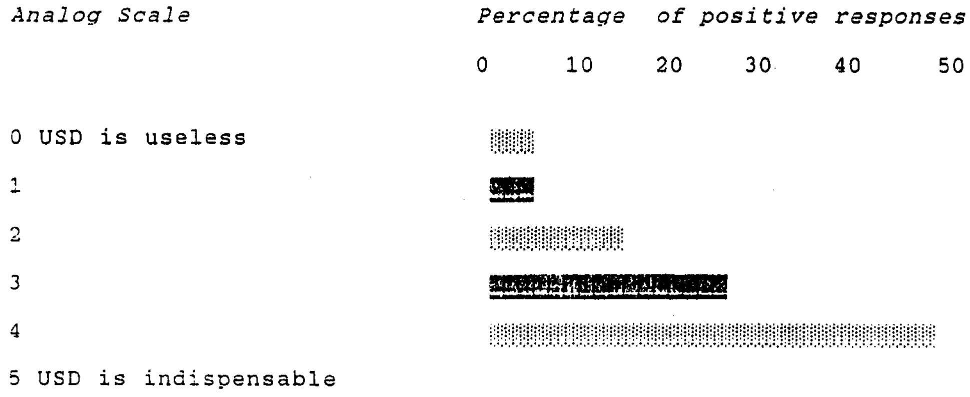

FIGURE 1

Surgeons' evaluation of usefulness of USD in hepatic resection (47 resections)

convenient. Similarly, the number of maneuvres required for the sterilization and assembly of the CUSA System handpiece, as well as the number of pieces to be assembled, prolonged the learning period and the time necessary to set up the equipment. Cleaning the handpiece after use took an average of 32 minutes for the CUSA System and 7 minutes for the NIIC-DX 101 T apparatus. Before sterilization of the handpiece, the CUSA System took 16 minutes to set up; the NIIC-DX

101 T device did not require any delay. The disposable tubing and bags of the CUSA system are presently four times more expensive than those of the NIIC-DX 101 T. The life span of the interchangeable tip is only one hour for the CUSA System compared with 300 hours for the NIIC-DX 101 T. As well, the size and weight of the CUSA System equipment is 125 kg compared with 80 kg for the NIIC-DX 101 T.

**Table 5** Time of required for operation and resection according to the type of hepatic resection performed*

| *Types of resection* | *No* | *Time required for opertion (min)* | *Time required for resection (min)* |
|---|---|---|---|
| Lobectomy | 10 | 352 (230–55) | 120 (45–240) |
| Left lateral segmentectomy | 8 | 307 (100–450) | 111 (40–200) |
| Other segmentectomies | 7 | 251 (90–480) | 107 (20–240) |
| Wedge resection | 10 | 241 (100–420) | 68 (30–130) |
| Cyst excision | 12 | 234 (150–360) | 95 (30–180) |

* mean (range)

### DISCUSSION

The USD facilitates formal hepatic resections. Access to intra and extraparenchymal arterial and venous tributaries and particularly the control of the hepatic veins are enhanced by USD. While transsection of hepatic parenchyma is neither easier nor more expedient than with conventional techniques, it appears less hemorrhagic. When the overall appraisal was expressed on an analog scale ranging from 0 to 5, the USD was found to be helpful or very helpful (grade 3 or 4) in 73 percent of resections. Total or partial excision of hydatid cysts was greatly facilitated by the use of the USD; this benefit was not found for wedge resections of other hepatic lesions. Converging opinions emerging from various surgical centers strengthen the conclusions of the present prospective survey, regardless of the technique, compared to use of the USD, or to underlying pathology.

Even though hepatic resection has become a standard procedure, it is still associated with significant morbidity and mortality. The most important postoperative complications include hepatic failure, abscess formation, and biliary fistulas. Hemorrhage constitutes the major operative complication[1–6] and intraoperative bleeding seems to be the most critical factor affecting the postoperative prognosis[6]. As the present series was not randomized, the volumes of blood and other fluids infused were thought to be indicative of the severity of the operation and underlying disease, rather than related to the use of one or the other devices. In the surgeon's opinion, parenchymal section was less hemorrhagic with the USD (Table 3). In fact this evaluation is partly subjective as bleeding can be masked by the continuous aspiration of the USD. Nevertheless, infused blood volumes are objective measurements and seem to sustain this opinion. Although blood volumes infused during operation may differ slightly from true blood loss, our results (Table 2) can be compared with other experiences with or without the USD. Estimated blood losses of 5.5 L to 2.5 L have been quoted for major hepatic resections without the USD [4–6]. In Hodgson's experience with the USD[10], the average blood loss was 1.580 L for lobectomies, 0.644 L for left lateral segmentectomies, and 0.75 L for wedge resections. For the same procedures the volumes of blood infused in our study were 1.9 L, 1.1 L, and 0.9 L, respectively (Table 2). The mean blood loss reported by Andrus *et al.*[12] was 0.8 L for 13 segmentectomies with the USD; the mean volume of blood infused for the same type of operation was similar in our study (0.8 (range 0.0–2.4) L). One advantage of our multicenter study, especially since part of the criteria are subjective, is to provide information which does not depend on a single surgeon or surgical center. Indeed, as previously mentioned, this study was not randomized. To our knowledge, the only randomized study on the subject is the experience of the National Cancer Institute (NCI), reported briefly by Adson in the discussion following the above-mentioned article of Andrus *et al.*[12]. Two NCI surgeons, after they became acquainted with the device, randomized 11 patients to undergo hepatic resections with or without the USD. The mean blood loss was 1.7 L when the dissector was used, compared with 3.0 L when the dissector was not used. Four other surgeons at the same institution refused to give up the use of the USD to participate in the study.

No intraoperative deaths were recorded in the present survey. Our perioperative figures of 4.8% mortality and 12% morbidity compare favorably with those of other series[4–6]. Postoperative hemorrhage, abscess formation, and biliary leaks resulted either from inadequate control of bleeding, or extensive devitalization of tissues at the site of parenchymal resection[3]. One of the theoretical advantages of the USD is to provide optimal control of bleeding during the dissection of the liver parenchyma, reducing the amount of devitalised tissue when compared with other techniques[14]. However the reduction of devitalized residues is also influenced by rigorous attention to the segmental anatomy of the liver and avoidance of transfixing mattress sutures at the margin of resection. The benefits of omental[15] or other flaps[16] and/or of various local hemostatic substances to this end warrant further investigation.

The major advantage of the USD which became apparent during this study was the ease of control of arterial and venous intra- and extrahepatic pedicles, with particular reference to the hepatic veins. On the other hand, the parenchymal section was found to be neither easier nor more expedient than with the more commonly used techniques. Andrus *et al.*[12], utilizing the USD in 13 patients, stressed that transection of the hepatic parenchyma using the USD was tedious and slow as compared with the finger or clamp fracture techniques. The duration of operation or resection (Table 5) may be related to the initial disease and to the importance of the procedure performed. In this study, the duration of resections was measured from the start of dissection until achievement of complete hemostasis. Comparison with other reports is rather confusing because of the lack of a strict definition of duration of resection. The mean duration required to perform segmental resections was $128 \pm 57$ min in Andrus' experience (2) and $107 \pm 81$ min in our study. The overall impression of surgeons using this device, expressed on an analog scale at the end of the operation, was good or very good in approximately

75% of cases. Furthermore, it seemed obvious for the surgeons who had the opportunity to use the dissectors more than twice, that the rating improved with the number of resections performed. As compared to the other types of resection and with respect to the other possible methods - fracture technique or electrocautery - major hepatectomies, segmental resections, and total and partial excisions of cysts were facilitated by the USD. For major hepatectomies this benefit is most likely due to the ease and security of control of large vessels. For segmental resections, the USD allows sparing of hepatic parenchyma, i.e. more selective resections than would have been imposed by primary control of portal and arterial blood supply. Utilisation of ultrasonic dissection facilitates completion of segmental hepatic resection without need of temporary occlusion of the vascular structures at the porta hepatis. The benefits in cyst excision observed in the present study have never been mentioned before in the evaluation of the ultrasonic surgical dissectors. In fact, when total and partial ablations of cysts were performed as a single procedure, no intraoperative blood transfusion was required at all.

Although all surgeons did not have the chance to compare the two types of USD, there seemed to be a tendency to prefer the NIIC-DX 101 T to the CUSA System, based on the user friendliness, and the facility of maintenance and upkeep. As well, cost-effectiveness was in favor of the NIIC-DX 101 T device.

In addition to use in the fields of ophthalmology[17] and neurosurgery[18], the USD has also been employed in urology[19], partial splenectomy[20], as well as for various other general surgical procedures[21–23]. The largest experimental and clinical series published to date, however, deal with hepatic surgery[10,12,13]. Other techniques have been suggested to assist in hepatic resection, which may either be compared to USD, for instance the water-jet[9], or used in conjunction with the device, to improve operating conditions, such as the ND-YAG laser[8] or the microwave tissue coagulator[7]. The particular aim of the latter is to achieve liver tissue hemostasis, and this technique may prove to be a useful adjunct to USD, particularly with reference to segmental resection. Because of the high costs of these devices, however, and especially when used conjointly, further prospective studies are warranted before advocating their routine use.

### *References*

1. Foster, J.H. (1978) Survival after liver resection for secondary tumors. *Am. J. Surg.*, **135**, 389–394
2. Lee, N.W. and Ong, G.B. (1982) The surgical management of primary carcinoma of the liver. *World J. Surg.*, **6**, 66–75
3. Ryan, W.H., Hummel, B.W. and McClelland, R.N. (1982) Reduction in the morbidity and mortality of major hepatic resections. *Am. J. Surg.*, **144**, 740–743
4. Thompson, H.H., Tompkins, R.K. and Longmire, W.P. (1983) Major hepatic resection. A 25-year experience. *Ann. Surg.*, **197**, 375--388
5. Iwatsuki, S., Shaw, B.W. and Starzl, T.E. (1983) Experience with 150 liver resections. *Ann. Surg.*, **197**, 247–253
6. Nagao, T., Inoue, S., Mizuta, T., Saito, H., Kawano, N. and Morioka, Y. (1985) One hundred hepatic resections. Indications and operative results. *Ann. Surg.*, **202**, 42–49
7. Tabuse, K., Katsumi, M. and Kobayashi, Y. *et al.* (1985) Microwave surgery: Hepatectomy using a microwave tissue coagulator. *World J. Surg.*, **9**, 136–143
8. Joffe, N.S., Brackett, K.A., Sankar, M.Y. and Daikuzono, N. (1986) Resection of the liver with the ND:YAG Laser. *Surg. Gynecol. Obstet.*, **163**, 437–442
9. Dimitrios, N., Papachristou, N. and Barters, R. (1982) Resection of the liver with a water jet. *Br. J. Surg.*, **69**, 93–94
10. Hodgson, J.B. and DelGuercio, L.R.M (1984) Preliminary experience in liver surgery using the ultrasonic scalpel. *Surgery*, **95**, 230–234

11. Putnam, C.W. (1983) Techniques of ultrasonic dissection in resection of the liver. *Surg. Gynecol. Obstet.*, **157**, 474–478
12. Andrus, C.H. and Kaminski, D.L. (1986) Segmental hepatic resection utilizing the ultrasonic dissector. *Arch. Surg.*, **121**, 515–521
13. Thomson, S.R., Francel, T.J. and Youngson, G.G. (1987) Cavitron assisted liver resection in a child. *J. Pediatr. Surg.*, **22**, 363–364
14. Tranberg, K.G., Rigotti, P., Brackett, K.A., Bjornson, H.S., Fischer, J.E. and Joffe, S.N. (1986) Liver resection. A comparison using the Nd-YAG laser, an ultrasonic surgical aspirator, or blunt dissection. *Am. J. Surg.*, **151**, 368–373
15. Stone, H. and Lamb, J. (1975) Use of pedicled omentum as autogenous pack for control of hemorrhage in major injuries of the liver. *Surg. Gynecol. Obstet.*, **141**, 92–94
16. Didolkar, M.S. and Fitzpatrick, J.L. (1986) Gerota's fascia flap for control of hemorrhage of the hepatic surface. *Surg. Gynecol. Obstet.*, **163**, 485–486
17. Kelman, C.D. (1973) Phaco emulsification and aspiration. A report of 500 consecutive cases. *Am. J. Ophtalmol.*, **75**, 764–768
18. Flamm, E.S., Ransohoff, J., Wunchinich, D. and Broadwin, A. (1978) Preliminary experience with ultrasonic aspirator in neurosurgery. *Neurosurgery*, **2**, 240–245
19. Addonizio, J.C., Choudhury, M.S. Sayegh, N. and Chopp, R.T. (1984) Cavitron ultrasonic surgical aspirator: applications in urologic surgery. *Urology*, **23**, 417–420
20. Hodgson, J.B. and McElhinney, A.J. (1982) Ultrasonic partial splenectomy. *Surgery*, **91**, 346–348
21. Heimann, T.M., Kurtz, R.J. and Aufses, A.H. (1985) Ultrasonic fragmentation. A new technique for mucosal protectomy. *Arch. Surg.*, **120**, 1200–1203
22. Hodgson, J.B., Poddar, P.K., Mencer, E.J., Williams, J., Drew, M. and McElhinney, A.J. (1979) Evaluation of ultrasonically powered instruments in the laboratory and in the clinical settings. *Am. J. Gastroenterol.*, **72**, 133–140
23. Hodgson, J.B. (1979) The ultrasonic scalpel. *Bull. NY Acad. Sci.*, **55**, 908–915

*(Accepted by S. Bengmark 16 September 1991)*

### INVITED COMMENTARY

Millat *et al.*, in this paper have produced the first prospective multicenter study to determine if ultrasonic dissectors (USD) were useful in hepatic resection. Individual subjectivity was reduced by using multiple surgeons. But since each surgeon did a small number of cases, it could be argued that their experience will be biased because they were neophytes with the device. However, the learning curve appeared to be short since it was noted that those surgeons who used the USD more than twice felt more and more comfortable with each case performed.

In 1986 Andrus studied the USD in thirteen patients, but found that hepatic transection was tedious and slow compared with the finger fracture or the clamp fracture technique. This cry has been taken up by several very experienced hepatic surgeons who are used to rapidly dissecting through hepatic parenchyma and then spending a good deal of time controlling bleeding afterwards.

Nonetheless, because the end result is so much cleaner and dryer with the USD, very little time is required at the end in order to control bleeding and I have always felt that it was worth the extra time. Furthermore, over the years, as experience has been gained, it no longer seems to be valid to claim that the USD slows down hepatic surgery. In fact, Millat *et al.* were much quicker than Andrus.

Several studies including this paper, show that blood loss is reduced in hepatic surgery when the USD was used. Andrus lost 0.8 L for segmentectomies; Millat lost 1.9 L for lobectomies, 1.1 L for left lateral segmentectomies, and 0.9 L for sub-segmental resections. Little and Hollands compared two groups of patients. Those in whom the CUSA was used had a median blood loss of 450 ml. In their control group, without the CUSA, blood loss was tripled. I recently examined 33 patients who between them had 37 tumors, averaging 5.6 cm in size. There were 5 right trisegmentectomies, 12 lobectomies, 15 segmental resections, and 4 sub-segmental resections with an average blood loss of only 1.0 l per case.

Little and Hollands used portal clamping but Millat *et al.* used it rarely. I also use portal clamping up to 45 minutes at a time. This I believe allows more meticulous surgery and a very dry field and probably reduces the blood loss by half as much again. The risk of blood transfusion is therefore reduced. The very dry field also possibly is a factor in reduction in the complication rate. Because of these points, hospital stay can be reduced and this reduction could help offset the cost of the USD.

Clearly, the USD does not a surgeon make. However, for those who are interested, it does allow them to find hepatic anatomy once the effected segment has previously been fully mobilized. It is my hope that more surgeons will be able to safely perform hepatic resections because of the USD and thus more patients will benefit and the cycle of referrals will be increased.

### *References*

Andrus, C.H. and Kaminski, D.L. (1986) Segmental hepatic resection utilizing the ultrasonic dissector. *Arch. Surg.*, **121**, 515–521

Little, J.H. and Hollands, M.J. (1991) Impact of the CUSA and Operative Ultrasound on Hepatic Resection. *HPB Surg.*, **3**, 171–178

Hodgson, W.J.B., Morgan, J., Byrne, D. and DelGuercio, L.R.M. Hepatic Resections for Primary and Metastatic Tumors using the Ultrasonic Surgical Dissector (CUSA$^R$). *Amer. J. Surg.*, (in press).

John B. Hodgson
Department of Surgery
Munger Pavillion
New York Medical School
Valhalla, New York 10595
USA

